# The impact of hydroxyapatite crystal structures and protein interactions on bone's mechanical properties

**DOI:** 10.1038/s41598-024-60701-7

**Published:** 2024-04-29

**Authors:** Yadi Sun, Yan Wang, Chunhui Ji, Jianxiong Ma, Bingnan He

**Affiliations:** 1 Tianjin Hospital, Tianjin University, Tianjin, 300211 People’s Republic of China; 2 School of Mechanical Engineering, Tianjin University, Tianjin, 300072 People’s Republic of China; 3 Tianjin Orthopedic Institute, Tianjin, 300050 People’s Republic of China; 4 Tianjin Key Laboratory of Orthopedic Biomechanics and Medical Engineering, Tianjin, 300050 People’s Republic of China

**Keywords:** Molecular biology, Materials science

## Abstract

Hydroxyapatite (HAP) constitutes the primary mineral component of bones, and its crystal structure, along with the surface interaction with proteins, significantly influences the outstanding mechanical properties of bone. This study focuses on natural hydroxyapatite, constructing a surface model with calcium vacancy defects. Employing a representative model of aspartic acid residues, we delve into the adsorption mechanism on the crystal surface and scrutinize the adsorption forms of amino acid residues on HAP and calcium-deficient hydroxyapatite (CDHA) surfaces. The research also explores the impact of different environments on adsorption energy. Furthermore, a simplified sandwich structure of crystal-polypeptide-crystal is presented, analyzing the distribution of amino acid residue adsorption sites on the crystal surface of the polypeptide fragment. This investigation aims to elucidate how the stick–slip mechanism of polypeptide molecules on the crystal surface influences the mechanical properties of the system. By uncovering the interface mechanical behavior between HAP and osteopontin peptides, this article offers valuable theoretical insights for the construction and biomimetic design of biocomposites.

## Introduction

Bone, a paradigmatic nanocomposite material, is composed of 25% proteinaceous organic matter and primarily features Hydroxyapatite (HAP) crystals in its inorganic components^1^. Remarkably, the crystal structure undergoes dynamic alterations within living organisms. The mineralization process of HAP in natural bone tissue unfolds in a low-temperature and body fluid environment, resulting in the formation of CDHA due to calcium vacancy defects^2,3^. During the formation of CDHA, protons in the solution environment bind with hydroxyl and phosphate groups to form H_2_O and HPO_4_^2^, leading to increased calcium precipitation and formation of calcium vacancies in the crystal; ultimately, Ca II vacancies are replaced by adjacent H_2_O and HPO_4_^2−^ to maintain charge neutrality, resulting in the formation of calcium-deficient hydroxyapatite in bone tissues^4^. In the solution environment of CDHA, proton interactions lead to the formation of water and hydrogen phosphate ions, causing a shift in the Ca/P ratio as the pH decreases^5,6^. The correction of calcium vacancies by protons results in the formation of crystals with a chemical formula of Ca_10−x_(HPO_4_)_x_(PO_4_)_6−x_(OH)_2−x_ (0 < x < 1)^7^.

Natural apatite in bone tissue, particularly CDHA formed through organism metabolism, significantly contributes to bone reconstruction and growth, imparting strength and supporting physiological activities^8,9^. Despite this, the mechanical properties, including bone toughness, are intricately linked to the organic component's proteins^10^. Through an examination of bone tissue microstructure, it is evident that osteopontin (OPN) acts as a binder at the crystal-protein interface, promoting strong collagen adhesion to the crystal surface and enhancing bone toughness^11,12^. OPN presence reduces structural deformation and fractures in collagen and mineral crystals in the staggered arrangement of bones, also mitigating the loss of interface adsorption energy during bone damage^13^. Highly phosphorylated OPN, rich in aspartic and glutamic acid, demonstrates diverse regulatory functions concerning HAP crystals^14^. The analysis of OPN's amino acid residues reveals its robust binding ability to calcium ions, particularly on the crystal's (100) surface^15–17^. Studies modifying OPN's conformation through phosphorylation enhance its binding affinity to the HAP surface^18–21^. Amino acid residues containing carboxyl groups, like aspartic acid and glutamic acid, play a pivotal role in the interaction between proteins and crystals, as confirmed by molecular dynamics simulations of OPN adsorption^22,23^.

In the intricate process of bone formation, HAP crystals embark on their initiation and development journey within the periodic interstitial channels nestled between adjacent collagen molecules^24,25^. Over time, a prevailing perspective suggests that these crystals adopt a staggered arrangement, contributing to a structural framework endowed with exceptional toughness^26^. This resilience is primarily attributed to the synergistic effects of proteins releasing fracture energy and the adsorption energy at the two-phase interface^27,28^. At the nanoscale, the interface interaction between HAP crystals and collagen showcases remarkable resistance to high shear loads, underscoring the imperative need to explore interface mechanical behavior for a comprehensive understanding of bone tissue material properties^29^.

Within bone tissue, the intricate cross-linking between organic matter and crystals is not solely dictated by adsorption sites but is intricately influenced by factors like the surface morphology of crystals and the dynamic sliding of other protein molecules on the crystal surface^30^. The biomolecular regulation during HAP nucleation and growth induces calcium vacancy defects, prompting alterations in both internal and surface spatial structures, introducing irregularities and undulations in the crystal lattice^31^. The atomic morphology on the HAP surface plays a pivotal role in influencing protein adsorption capacity, with research indicating that the concave and convex structure of the crystal surface enhances protein interaction^32^. However, the characterization of the surface structure based on calcium ion morphology remains an underexplored realm. Moreover, at the nanoscale, shear load governs slip at the interface between protein molecules and crystals, dissipating a substantial amount of energy and efficaciously averting material failure^33^. The resistance to slip at this interface is multifactorial, encompassing considerations of the viscoelastic properties of proteins, interface adsorption energy, adsorption form, among others.

In this study, we delve into the nanoscale examination of mineralized collagen fibers in bones. During crystal nucleation and growth, HAP displays a specific orientation influenced by the body fluid environment, revealing internal flaws. Limited research exists on the adsorption site mechanism during crystal-protein interaction, particularly in understanding the unclear interface toughening mechanism. Employing molecular dynamics, we constructed comprehensive surface models of HAP (100) and CDHA (100) surfaces. Molecular dynamics simulations investigated the interaction mechanism of aspartic acid residues on the crystal surface, aiming to unveil the protein adsorption site interaction and explore the impact of different environments on adsorption energy. Additionally, crystal models with diverse surface morphologies were examined to understand their influence on the interface behavior of ossification protein polypeptide chains under shear load through molecular dynamics restricted shear simulation. This effort aims to elucidate biological responses under shear load and the anti-deformation mechanism of composite materials, providing theoretical guidance for biocomposite construction and biomimetic design.

## Materials and methods

### Modeling description

**Figure 1:**
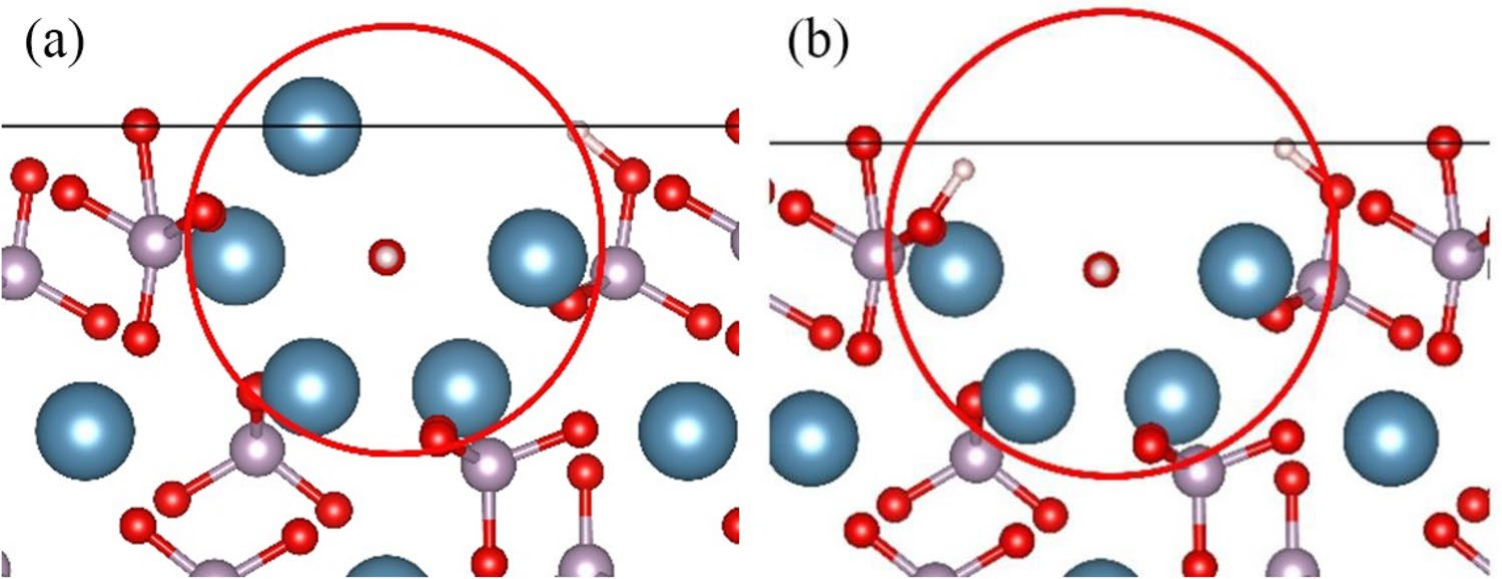
Ca II vacancy model: (**a**) CDHA-1, and (**b**) CDHA-2.

HAP is represented as Ca_10_(PO_4_)_6_(OH)_2_ with P63/m symmetry, having lattice parameters *a* = *b* = 0.9432 nm, *c* = 0.6881 nm, and angles *α* = *β* = 90°, *γ* = 120°. The crystal contains two types of Ca atoms: Ca I (4 atoms) at *z* = 0 and *z* = 1/2, and Ca II (6 atoms) forming a channel structure along the c-axis, creating a triangular arrangement around hydroxyl groups^34–36^. The CDHA is delineated by the formula Ca_10−x_(HPO_4_)_x_(PO_4_)_6−x_(H_2_O)_x_ (0 < x ≤ 1). Given that the mineralization process of biological bone tissue takes place in the relatively lower-temperature environment of body fluids and is regulated by acidic amino acids^37^, it is more probable for HAP containing Ca II vacancies to form in bone tissue. Therefore, this work focuses on HAP crystals with Ca II vacancy defects. In the model, one Ca II atom is removed, and the charge is compensated by two protons. The removed Ca II atom is then combined with adjacent PO_4_^3−^ groups and hydroxyl groups, respectively, forming HPO_4_^2−^ groups. In this work, two forms of defect structures were constructed, as illustrated in Fig. 1. These structures are identified as the single defect structure CDHA (CDHA-1) and the double defect structure CDHA (CDHA-2).

**Figure 2:**
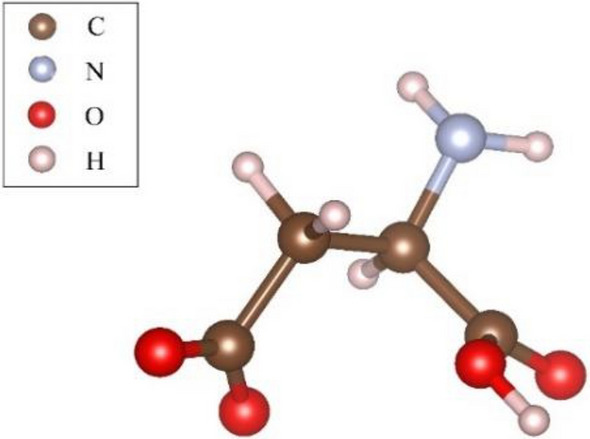
Aspartic acid residue model.

The investigation into adsorption sites focused on aspartic acid residues as the subject of study^38^. Aspartic acid, with a chemical formula of HOOCCH_2_CH(NH_2_)COOH, is known for its easy combination with calcium ions to form adsorption sites. The potential energy of aspartic acid is described using COMPASS II force field parameters^39^. The geometric optimization of the aspartate structure involves ensuring that the initial aspartate residue attains the most stable structural state with the smallest initial potential energy. The optimized spatial structure is illustrated in Fig. 2.

**Figure 3:**
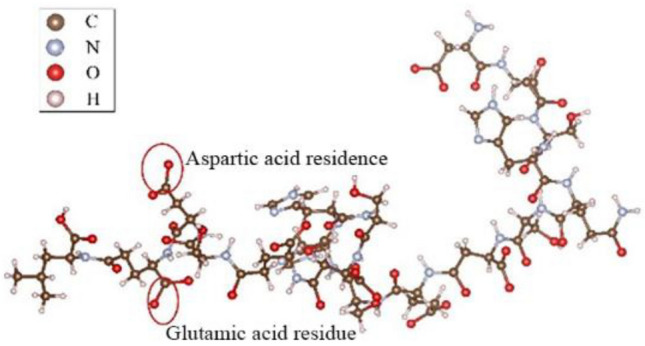
OPN peptide model.

OPN in humans comprises 298 amino acid residues, with approximately 25% of them being acidic amino acids. Drawing on existing research on OPN polypeptides, the amino acid sequence selected for this experiment is DDSHQSDESHHSDESDEL. The atomic structures of various amino acids are based on the research by Addison et al^40^. This polypeptide consists of 8 residues, each being either aspartic acid (D) or glutamic acid (E), and its structure is depicted in Fig. 3.

### Description of the method

In this study, we utilized the Forcite module within Materials Studio, a comprehensive computational platform for materials science developed by BIOVIA. The Forcite module is particularly adept at performing a range of computational tasks, including rapid energy calculations for single molecules and periodic systems, as well as geometric optimizations to identify stable conformations at minimum energy states. Additionally, Forcite supports molecular dynamics simulations, which are essential for understanding the dynamic behavior of molecules and their interactions within a given environment. For the purposes of this research, the Forcite module was employed for geometric optimization and molecular dynamics simulations to ensure that the modeled structures were in their energetically favorable states and to accurately capture the adsorption and shear behaviors of the materials under investigation.

### Adsorption simulation parameters

This study employs molecular dynamics simulation, placing the (100) face of two crystal types amid amino acid residues. The simulation starts from the optimized structural position, introducing 100 water molecules to hydrate the system and immobilizing atoms on the crystal surface. Two simulation environments are utilized: a neutral water molecule setting for comparison and an environment with added HPO_4_^2−^ and H_2_PO_4_^−^ in a 1:1 ratio. The Forcite module in Materials Studio is used for simulation, employing the NVT ensemble with Nosé-Hoover for temperature control to mimic the biological environment at 310 K. The Ewald method calculates electrostatic interactions, and the simulation has a time step of 1 fs, a system simulation time of 5 ps, and energy deviation controlled at 50,000 kcal/mol.

### Shear simulation settings

The restricted shear calculation in this study utilized the Forcite module in Materials Studio. Initially, the Amorphous Cell module constructed the polypeptide's amorphous structure, added to a simulation box (38 Å × 20 Å × 20 Å). The Build layer feature created a sandwich structure of crystals and polypeptides. The Forcite module's Confined Shear feature simulated with a shear speed of 1 Å/ps, a temperature of 310 K, a time step of 1 fs, and a total simulation time of 10 ps.

## Results and discussion

### Adsorption mechanism of aspartic acid residue on hydroxyapatite

**Figure 4:**
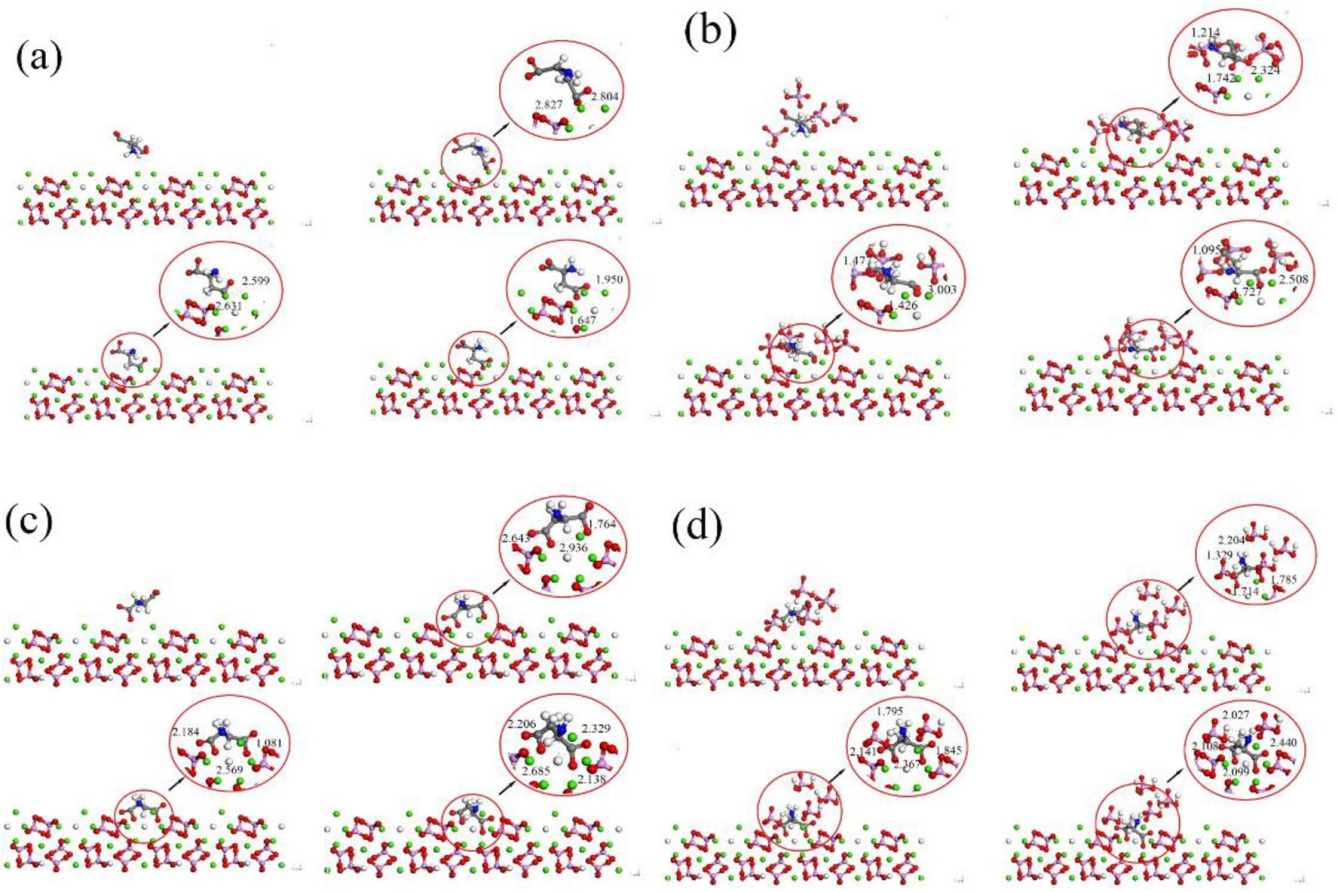
Atomic snapshots. (**a**) HAP (H_2_O). (**b**) HAP (HPO_4_). (**c**) CDHA (H_2_O). (**d**) CDHA (HPO_4_).

The molecular dynamics simulations were conducted to achieve the adsorption of aspartic acid residues on the (100) surface of both HAP and CDHA. However, there are differences in the adsorption behaviors between the two, and the atomic snapshots of the adsorption process are illustrated in Fig. 4.

In aqueous conditions, molecular dynamics simulations on the HAP surface reveal interactions between the carboxyl group of aspartic acid and calcium ions, forming close contacts with bond lengths ranging from approximately 1.6 to 2.5 Å. Additionally, the amino end of aspartic acid exhibits short-distance contacts with oxygen atoms of adjacent PO_4_^3−^ groups, forming bond lengths between 1.0 and 1.5 Å. By 3 ps, adsorption reaches a stable state, with both the amino and carboxyl groups extending outward. The carboxyl and amino groups elongate by 0.003 Å and 0.009 Å, respectively. In contrast, structural changes on the CDHA crystal surface are more significant, with carboxyl and amino groups extending by 0.009 Å and 0.011 Å, respectively, emphasizing the greater impact of vacancy defects on aspartic acid. In a hydrogen phosphate solution environment, structural changes in aspartic acid are more pronounced than in the aqueous solution.

Observing changes in aspartic acid and HAP during adsorption reveals that it mainly occurs through interactions of –COO (carboxyl) and NH_2_ (amino) groups. Calcium ions interact with –COO, and phosphate groups with NH_2_. The adsorption involves oxygen atoms on aspartic acid's carboxyl groups, carrying a negative charge, facilitating binding to Ca cations on the crystal surface. Additionally, the amino group tends to interact with the oxygen atom on the phosphate group. Oxygen atoms in carboxyl groups, having a larger charge, enhance electrostatic interactions. Adsorption between oxygen on the carboxyl group in aspartic acid and Ca ions on the HAP surface crucially strengthens the HAP-protein interface. In a phosphate buffer solution, the structural changes of aspartic acid are more pronounced compared to in water due to the acidic nature of the phosphate buffer. The acidic H_2_PO_4_^−^ ions in the solution protonate the functional groups of aspartic acid, forming NH_3_^+^ and –COOH_2_^+^ groups, leading to an overall positive charge on the molecule. This enhances internal electrostatic interactions and hydrogen bonding, making the molecule more compact and stable.

**Figure 5:**
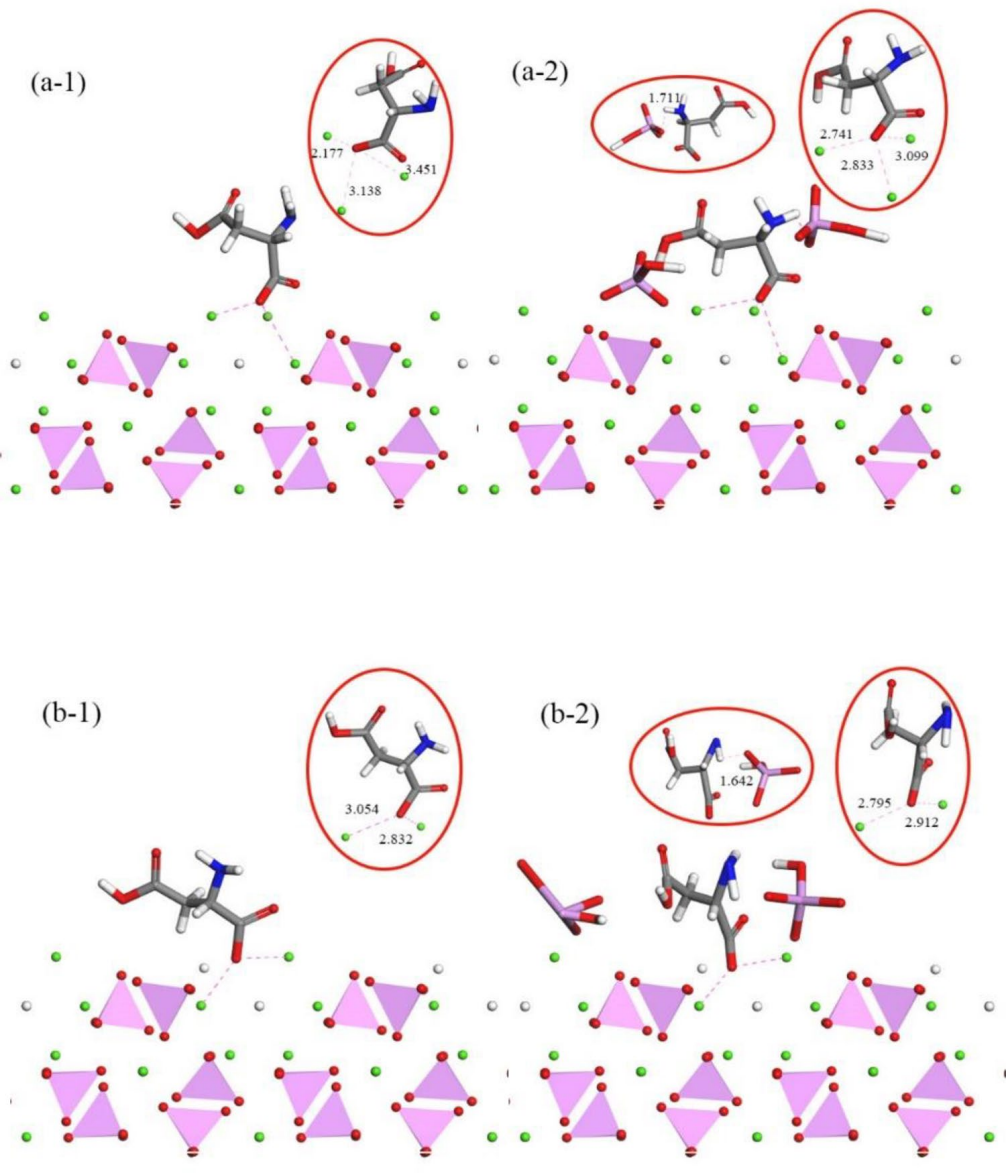
Interactions between –COO and Calcium Ion Channels: (**a-1**,**a-2**) depict the structure of the adsorption site on HAP, while (**b-1**,**b-2**) show the structure of the adsorption site on CDHA.

The examination of the adsorption process highlights the significant influence of the calcium triangular channel on the crystal, shaping the adsorption structure. Figure 5 displays the stable state and atomic snapshots, offering a visual understanding of –COO and calcium ion channel interactions.

In stable-state adsorption, carboxyl groups tightly bind to the HAP surface, with carboxyl group oxygen interacting with three calcium ions, maintaining a central position in a triangular structure. In vacancies, carboxyl groups shift closer to remaining calcium ions, reducing distances by 0.397 Å and 0.486 Å, positioning them near the crystal surface. Amino acid chain deformation reveals the amino group entering the space around HAP phosphate ions, forming hydrogen bonds with PO_4_ oxygen on the crystal surface. Amino end adsorption causes slight deformation in the corresponding carbon chain. During HAP mineralization, calcium vacancies induce structural changes, resulting in sparse HPO_4_ distribution on the surface, leading to weaker hydrogen bonding with the amino end. After adding hydrogen phosphate, HPO_4_ primarily interacts with aspartic acid's amino group. Hydrogen phosphate ionization imparts acidity, simulating HAP mineralization. Phosphate ions tend to adsorb on the crystal surface, mimicking growth along the (100) crystal plane, forming stronger hydrogen bonds than in a conventional solution environment.

### Interface behavior of polypeptide at the hydroxyapatite interface

**Figure 6:**
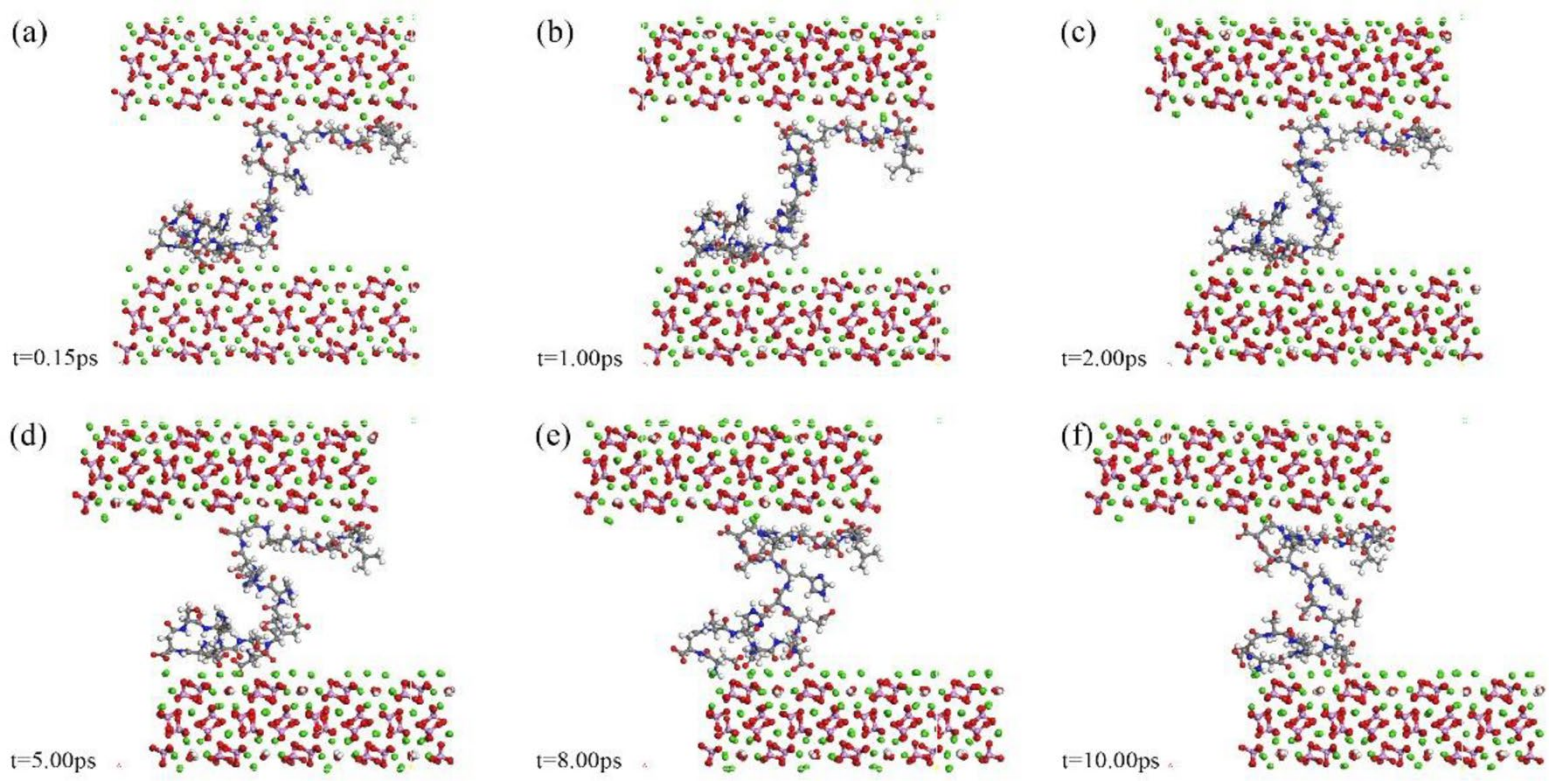
Atomic snapshots of deformation processes for HAP-peptide shear simulations.

**Figure 7:**
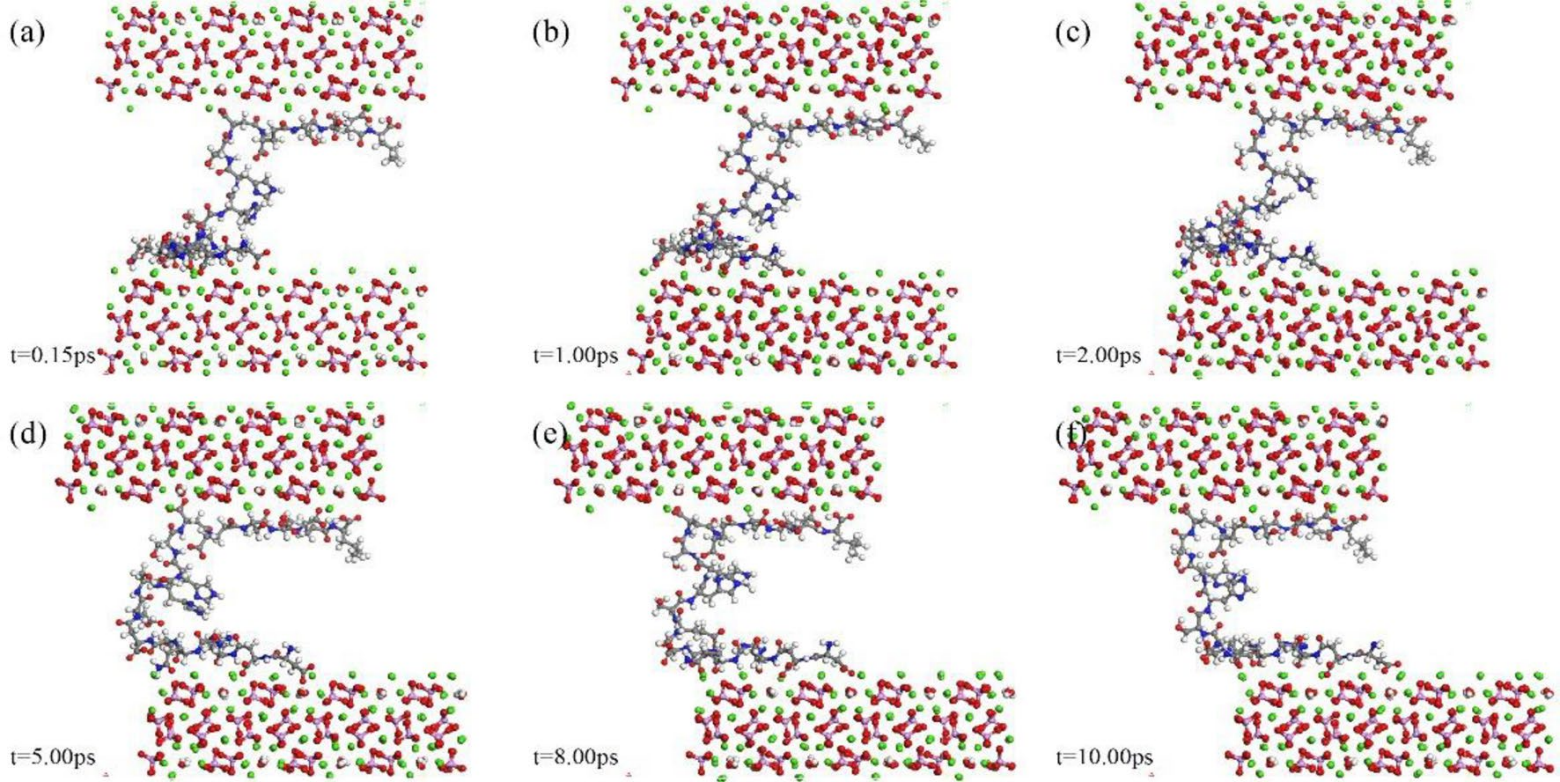
Atomic snapshots of deformation processes for CDHA1-peptide shear simulations.

**Figure 8:**
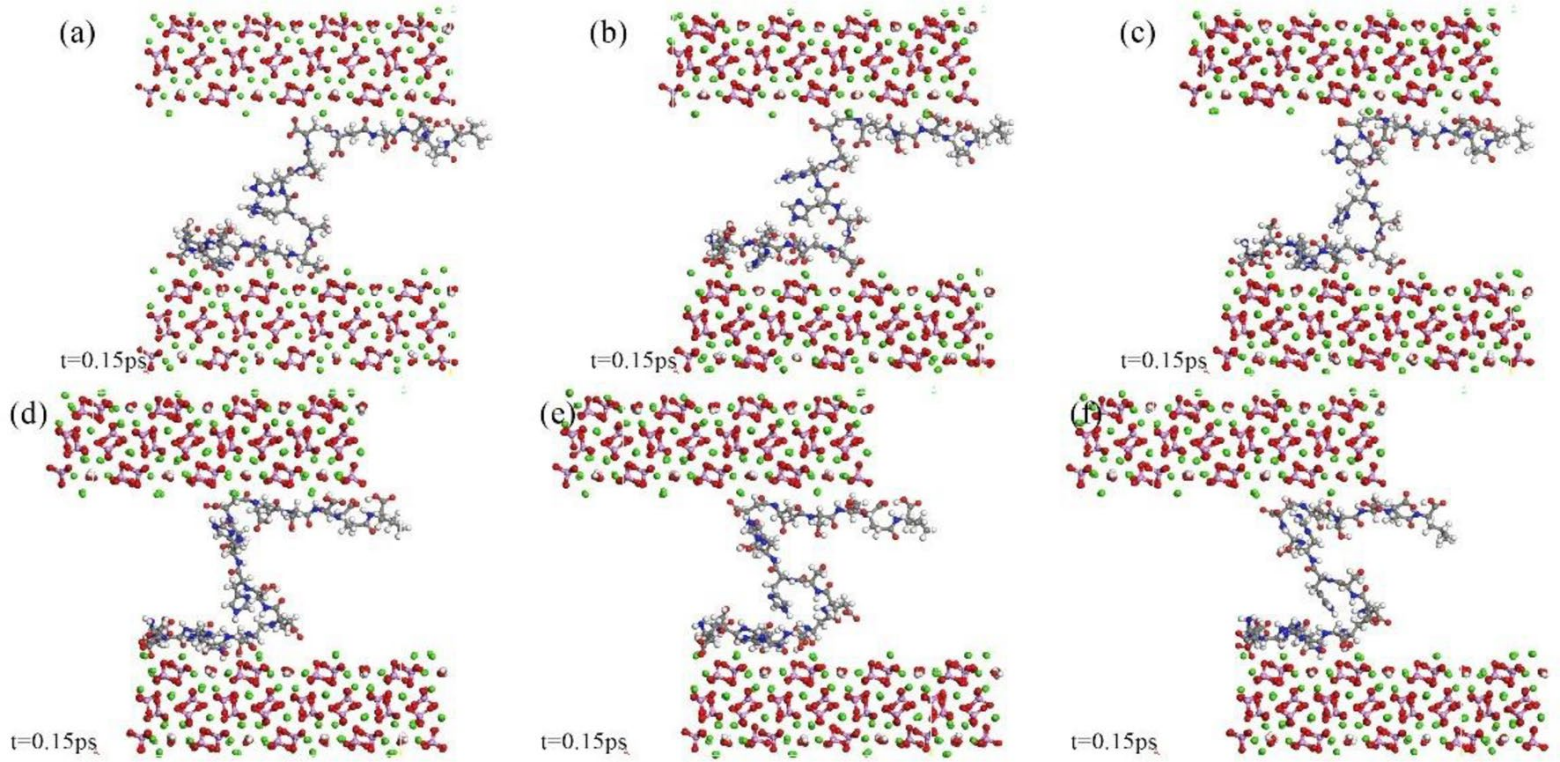
Atomic snapshots of deformation processes for CDHA2-peptide shear simulations.

Figures 6, 7 and 8 present atomic snapshots during shear simulations. Observing the first three snapshots (a), (b), (c), it is evident that the crystal's shear displacement is small, ranging from 0.15 to 2 Å. Consequently, there is limited structural change in the central polypeptide. Interface alterations primarily manifest in the configuration of peptide adsorption sites on the crystal. Amino acid residues on the peptide chain are concentrated at both ends, resulting in a "thicker at both ends, narrower in the middle" appearance. The following three snapshots (d), (e), (f) correspond to structural changes within 2–10 ps. Although there are minor energy fluctuations during this period, the overall trend is toward stability. The structure of residue aggregation areas at both ends of the peptide chain remains highly stable, undergoing minimal change during shear, indicating that the adsorption configuration of end residues has reached stability. Energy fluctuations are primarily caused by deformation in the spatial structure of the residue-free section in the middle of the peptide chain, with the transferred shear energy being compensated, ensuring the overall structural integrity of the simulation system.

**Figure 9:**
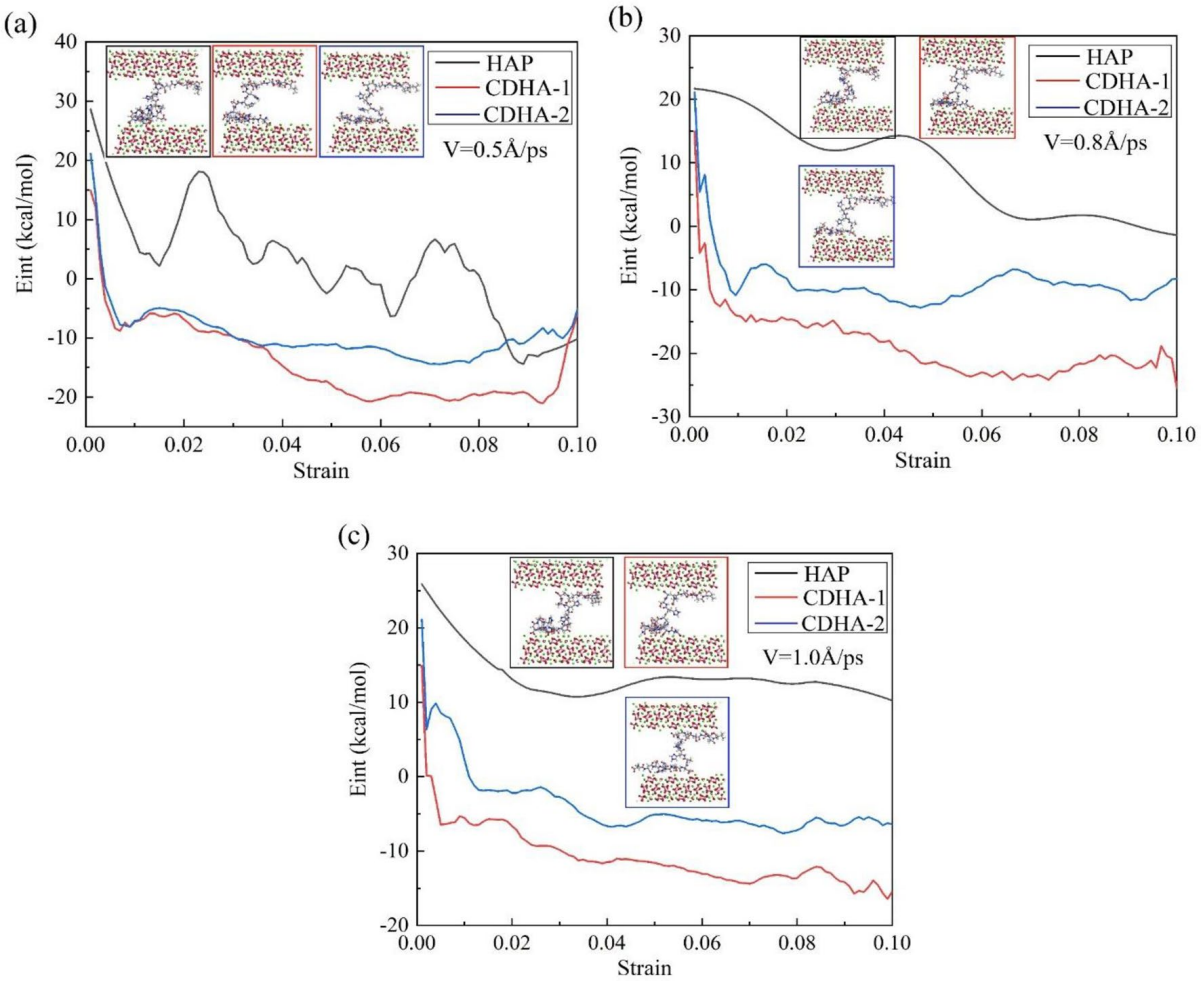
Shear Rate-dependent interaction energy and deformation processes.

This study further elucidates the interaction energy and deformation processes at different shear rates through atomic snapshots, as depicted in Fig. 9. The figure illustrates the interaction energy between the peptide chain and the upper and lower surfaces of HAP and two types of CDHA at shear rates of 0.5 Å/ps, 0.8 Å/ps, and 1.0 Å/ps, along with the structural features of the peptide and crystal when the configuration stabilizes. Among the three crystals, the interaction energy between HAP and the peptide is higher than the other two, but its stability is slightly compromised, showing greater fluctuations and requiring a longer loading process to reach a stable state. The interaction energy of HAP exhibits a staggered decreasing trend, while both CDHA types rapidly decrease to stable values before gradually stabilizing, a pattern similarly observed in (b) and (c). Furthermore, energy analysis reveals that, in the two CDHA crystal types, when the adsorption configuration reaches a stable state, the interaction energy between the single-vacancy crystal and the peptide is lower than that of the double-vacancy structure.

**Figure 10:**
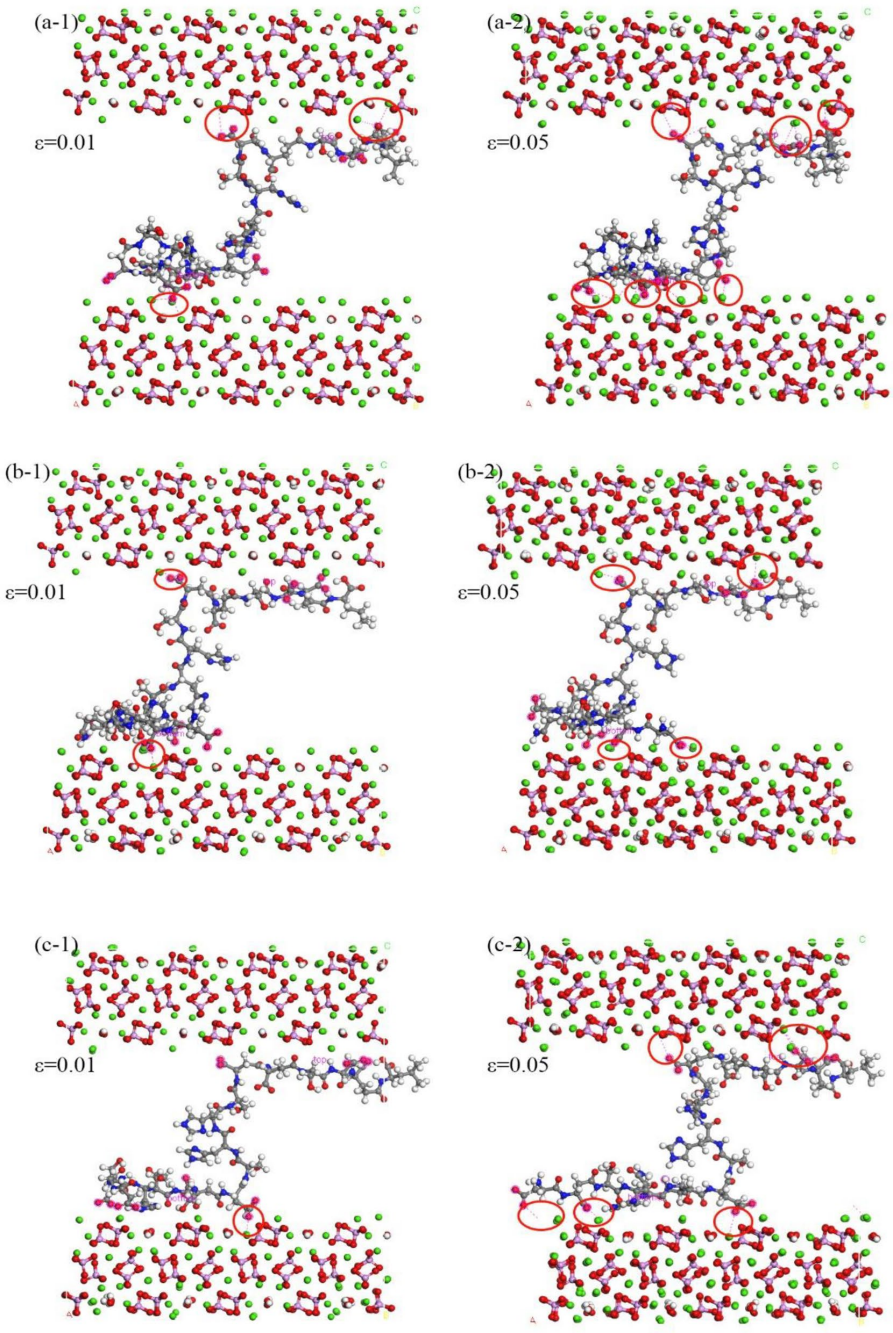
Shear-induced structural changes at the polypeptide-hydroxyapatite interface from 2-10 ps: (**a-1**) Initial Loading Phase and Reconstruction of Adsorbed Residues on HAP Interface. (**a-2**) Comprehensive Reconstruction of Adsorbed Residues on HAP Interface. (**b-1**) Initial Configurations of CDHA-1 with Vacancy Defects. (**b-2**) Stability Achieved in Adsorption Configurations of CDHA-1. (**c-1**) Initial Configurations of CDHA-2 with Vacancy Defects; (**c-2**) More Resilient Adsorption Configurations in CDHA-2.

Figure 10 intricately details the interface adsorption characteristics in simulation systems of HAP, CDHA-1, and CDHA-2 under a shear rate of 1.0 Å/ps, specifically focusing on strain values of 0.01 and 0.05. In the context of (a-1), the initial loading phase for the HAP crystal reveals a noteworthy reconstruction of adsorbed residues on the interface. Initially, a solitary amino acid residue (aspartic acid residue) on the lower interface significantly influences interface interactions, while the upper interface exhibits dual configurations. This dynamic is observed during the swift decrease in interaction energy between HAP and peptides as depicted in Fig. 9c. As the loading progresses, the configurations of adsorbed residues undergo a comprehensive reconstruction, as illustrated in (a-2). Nearly all residues participate in the adsorption process on both upper and lower interfaces, contributing to the stabilization of interaction energy. Analogously, in (b-1) and (c-1), configurations of structures with vacancy defects are initially fewer than those of HAP, elucidating their lower interaction energy. Subsequent to continued loading, the configurations on the interface achieve stability after reaching a strain value of 0.05, portrayed in (b-2) and (c-2). Notably, double-vacancy structures exhibit a proclivity for the aggregation of aspartic acid and glutamic acid residues on the peptide chain, resulting in more stable adsorption configurations in comparison to single-vacancy structures. Consequently, despite harboring a greater number of calcium ion vacancies, CDHA-2 manifests higher interaction energy and forms more resilient adsorption configurations compared to CDHA-1, a nuanced observation derived from Fig. 9.

## Conclusion

In conclusion, this study delves into nanoscale investigations of bone tissue, revealing insights into its mechanical properties and dynamic evolution. Employing comprehensive models and molecular dynamics simulations, the research analyzes stability changes during loading, emphasizing the pivotal -COO and Ca interaction and the influence of amino groups on aspartic acid's structural deformation. Calcium vacancies impact carboxyl group adsorption, and hydrogen phosphate stabilizes the process. The shear simulation highlights energy fluctuations and equilibrium attainment during adsorption, showcasing the efficiency of the double-vacancy structure in enhancing polypeptide adhesion and interface mechanical properties. Overall, the study provides theoretical guidance for biocomposite construction, bionic design, and microscale experiments.

## Data Availability

The data generated and/or analyzed during the current study cannot be publicly disclosed due to their intended use in subsequent experiments, but they are available upon reasonable request to the corresponding author.
